# Lysosome-Membrane Fusion Mediated Superoxide Production in Hyperglycaemia-Induced Endothelial Dysfunction

**DOI:** 10.1371/journal.pone.0030387

**Published:** 2012-01-12

**Authors:** Jun-Xiang Bao, Hui Chang, Yong-Gang Lv, Jin-Wen Yu, Yun-Gang Bai, Huan Liu, Yue Cai, Ling Wang, Jin Ma, Yao-Ming Chang

**Affiliations:** 1 Department of Aerospace Physiology, Fourth Military Medical University, Xi'an, People's Republic of China; 2 Department of Vascular and Endocrine Surgery, Xi Jing Hospital, Fourth Military Medical University, Xi'an, People's Republic of China; 3 Department of Aerospace Hygiene and Health Service, Fourth Military Medical University, Xi'an, People's Republic of China; Massachusetts General Hospital/Harvard Medical School, United States of America

## Abstract

Lysosomal exocytosis and fusion to cellular membrane is critical in the oxidative stress formation of endothelium under apoptotic stimulus. We investigated the role therein of it in hyperglycaemia-induced endothelial dysfunction. The lysosome-membrane fusion was shown by the expression of lamp1, the lysosomal membrane marker, on cellular membrane and the transportation of lysosomal symbolic enzymes into cultural medium. We also examined the ceramide production, lipid rafts (LRs) clustering, colocalization of gp91*^phox^*, a NADPH oxidase subunit (NOX) to LRs clusters, superoxide (O_2_^.^^-^) formation and nitric oxide (NO) content in human umbilical vein endothelial cells (HUVEC) and the endothelium-dependent NO-mediated vasodilation in isolated rat aorta. As compared to normal glucose (5.6 mmol/l, Ctrl) incubation, high glucose (22 mmol/l, HG) exposure facilitated the lysosome-membrane fusion in HUVEC shown by significantly increased quantity of lamp1 protein on cellular membrane and enhanced activity of lysosomal symbolized enzymes in cultural medium. HG incubation also elicited ceramide generation, LRs clustering and gp91*^phox^* colocalization to LRs clusters which were proved to mediate the HG induced O_2_^.^^-^ formation and NO depletion in HUVEC. Functionally, the endothelium-dependent NO-mediated vasodilation in aorta was blunted substantially after HG incubation. Moreover, the HG-induced effect including ceramide production, LRs clustering, gp91*^phox^* colocalization to LRs clusters, O_2_^.^^-^ formation and endothelial dysfunction could be blocked significantly by the inhibition of lysosome-membrane fusion. We propose that hyperglycaemia-induced endothelial impairment is closely related to the lysosome-membrane fusion and the following LRs clustering, LRs-NOX platforms formation and O_2_^.^^-^ production.

## Introduction

Cardiovascular complications such as atherosclerosis, myocardial infarction, and stroke etc are quite common in people with hyperglycaemia or diabetes. Endothelial injury or dysfunction is regarded as the most important initiating factor and earliest manifestation [1]. Among the mechanisms of endothelial dysfunction, oxidative stress or reactive oxygen species (ROS) generation is believed to play a crucial role [1], [2]. To our knowledge, elevated glucose levels could generate ROS by several pathways including nonenzymatic oxidation of glucose, increased mitochondrial oxidative phosphorylation, and increased activation of nicotinamide adenine dinucleotide phosphate (NADPH) oxidase etc [2], [3]. Simultaneously, the reduced activity of antioxidant defenses, such as alteration in antioxidant enzymes and decreased ascorbic acid levels may also accelerate endothelial impairment in hyperglycemic diabetic patients [2], [3].

As far as the NADPH oxidase is concerned, the activation of it depends largely on the assembling and aggregation of its subunits, including membrane-associated subunits gp91*^phox^* or its isoforms (NOX) and p22*^phox^*, as well as cytosolic subunits p47*^phox^*, p40*^phox^*, p67*^phox^*, and Rac [4], [5], [6]. Besides, the clustering of lipid rafts (LRs) has shown great promise to act as a driving force for the NOX assembling and aggregation [5], [6], [7]. LRs are unique micro-structures of membrane rich of cholesterol and lipids with saturated acyl chains, such as sphingolipids and glycosphingolipids. On stimulations, it would be clustered to form relatively large macrodomains serving to recruit or aggregate various receptors and signaling molecules in which NOX and other subunits of NADPH oxidase are listed [5], [6], [7]. Moreover, the LRs clustering has been proved to be promoted remarkably by the lysosomal exocytosis and fusion to cellular membrane [5], [6], [7].

As we have known, the function of lysosomes has not yet been restricted to breaking down waste materials and cellular debris. Some specialized lysosomal compartments storing newly synthesized secretory proteins have been found in several cell types which were referred to as secretory lysosomes or lysosome-related organelles [8], [9]. By lysosomal exocytosis and fusion with cellular membrane, inflammatory mediators, neurotransmitter, ATP, or glucagons could be transported outside the cell [8]–[15]. Beside, when the plasma membrane is damaged by mechanical, chemical or biological irritations, lysosomal membrane would act as a patch to do wound repair [16], [17], [18]. Lysosome-membrane fusion is also a pathway for some intracellular parasites to enter into and infect cell [8], [9], [19]. Recently, the lysosomal exocytosis and fusion to cellular membrane has also been found in bovine coronary arterial endothelial cells and glomerular endothelial cells, especially when the cells are under apoptotic stimulations [5], [6], [7], [20], [21], [22]. The acid spingomyelinase (ASM), a lysosomal hydrolytic enzyme, would be transported to cellular membrane and meet its substrate spingomylin by the lysosomal excytosis and induce the ceramide production there. In light of its biochemical and biophysical properties, ceramide molecules tend to be spontaneously self-associated and form small ceramide-enriched membrane microdomains which could contribute to the LRs clustering and then facilitate NOX aggregation and ROS generation [20], [21], [22].

As far as we are concerned, there is still no report on the lysosomal exocytosis and fusion in other types of endothelium, as well as no report on whether the hyperglycaemia or diabetes could induce lysosome-membrane fusion or LRs clustering. The role of them in high glucose induced ROS production or endothelial dysfunction is, as yet, largely unclear. Given the fact that hyperglycaemia could increase the intracellular free calcium concentration [23] and may lead to the damage of cellular membrane in endothelium [24], both of which are the triggers of lysosomal exocytosis and fusion in many cell types [8]–[11], [14]–[18], we hypothesis that in hyperglycaemia incubated human umbilical vein endothelial cells (HUVECs), lysosome-membrane fusion could happen resulting in ROS generation and endothelial dysfunction through LRs clustering and LRs-NOX platform formation.

## Results

### High glucose incubation induced the movement of lysosomal marker to cellular membrane

**Figure 1:**
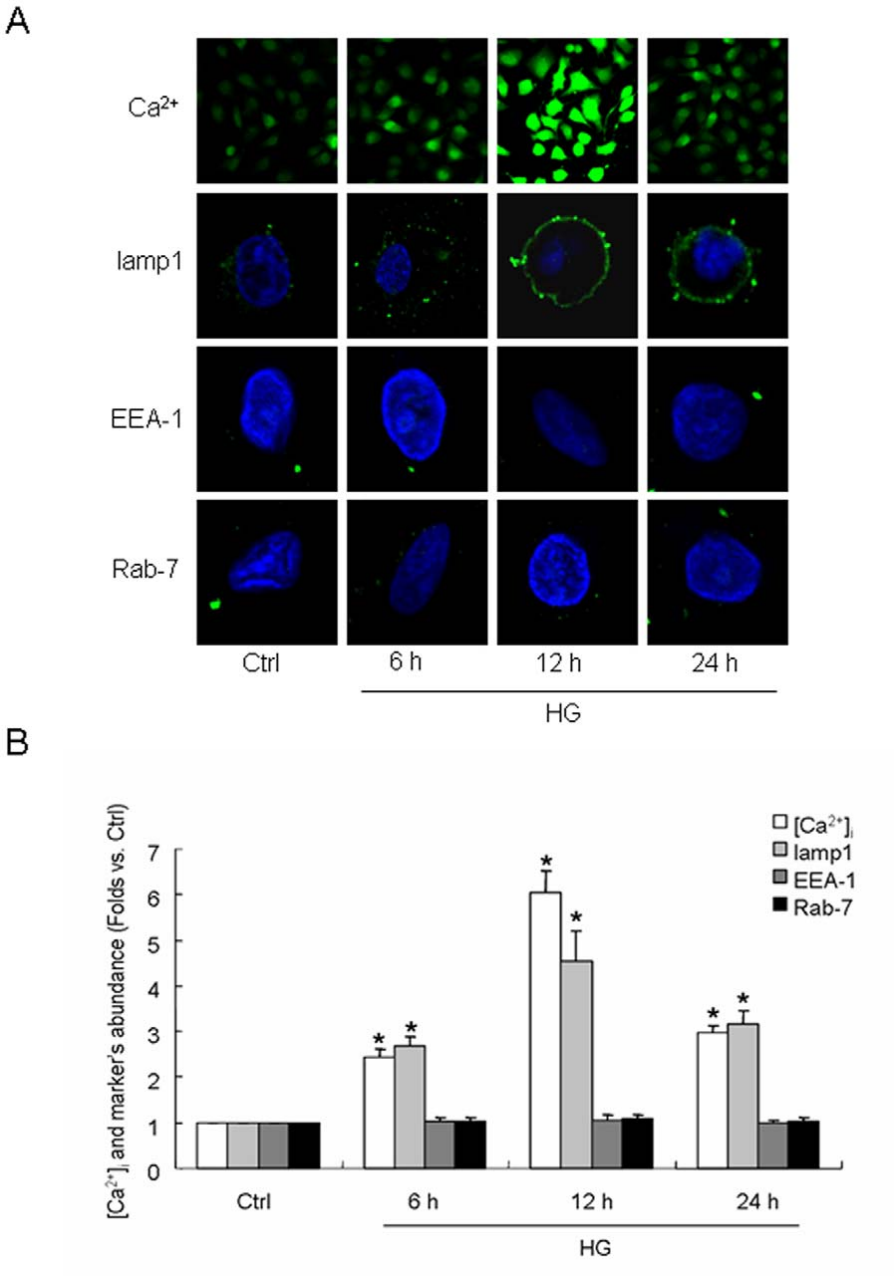
Lysosomal moving to cellular membrane promoted by hyperglycemia. A: representative images of intracellular free Ca^2+^ concentration ([Ca^2+^]_i_) and presentation of lamp1, EEA-1 and Rab-7, the marker of lysosome, early endosome and late endosome respectively, on membrane of human umbilical vein endothelial cells (HUVEC) stimulated by normal glucose (5.6 mmol/l, Ctrl) or high glucose (22 mmol/l, HG) for 6 h, 12 h and 24 h. B: summarized data of [Ca^2+^]_i_ and fluorescent abundance of lamp1, EEA-1 or Rab-7 on cellular membrane in different groups. Values are means±SE; n = 5 batches of cells. * P<0.05 vs. Ctrl.

To investigate the effects of high glucose incubation on lysosomal fusion to cellular membrane, the cells were cultured in the medium with normal glucose (5.6 mmol/l, Ctrl) or high glucose (22 mmol/l, HG) for 6 h, 12 h or 24 h. The intracellular free calcium concentration ([Ca^2+^]_i_) and fluorescence of lamp1, EEA-1 and Rab-7, which are the marker of lysosome, early endosome and late endosome respectively, on cellular membrane were detected. As shown in the confocal images (A) and summarized data (B) of Figure 1, [Ca^2+^]_i_ in cell with HG incubation for 6 h, 12 h or 24 h increased 2.43, 6.05 or 2.97-fold compared with that in Ctrl cell (P<0.05). Consistently, the abundance of lamp1 fluorescence on membrane of cell with HG incubation for 6 h, 12 h or 24 h was also higher significantly than the control level (2.68, 4.55 or 3.15-fold, P<0.05). However, neither EEA-1 nor Rab-7 was discovered to be present distinctly on membrane of both Ctrl and HG cell. We also detected the [Ca^2+^]_i_ and expression of lamp1, EEA-1 and Rab-7 on mannitol incubated cell and got the similar result as that in Ctrl cell (data not shown) which means osmotic pressure was not the cause for the change of [Ca^2+^]_i_ and lamp1 fluorescence on cellular membrane shown in HG cell.

### HG-induced movement of lamp1 to cellular membrane was prevented by lysosomal inhibitors or ASM small interfering RNA (siRNA) transfection

**Figure 2:**
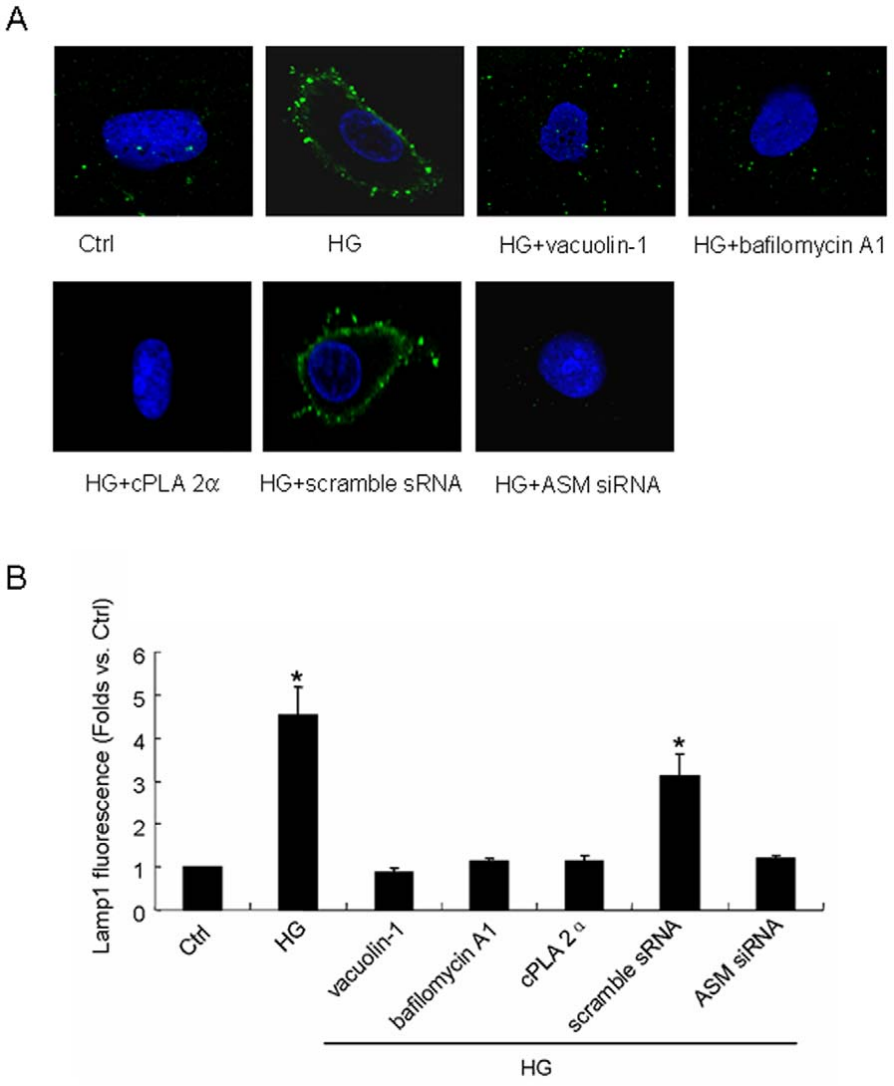
Effect of lysosomal inhibitors or acid sphingomyelinase (ASM) gene silencing on HG-induced movement of lamp1 to cellular membrane. A: representative images of lamp1 presentation on cellular membrane. The cells were cultured in normal glucose (5.6 mmol/l, Ctrl) or high glucose (22 mmol/l, HG) medium for 12 h with or without the pretreatment by vacuolin-1(10 µmol/l), bafilomycin A1 (100 nmol/l) or cPLA 2α (20 µmol/l) for 1 h or by the transfection of scramble small RNA (sRNA) or ASM small interfering RNA (siRNA). Then the lamp1 fluorescence on cellular membrane were detected and analyzed. B: summarized results of lamp 1 fluorescent abundance on cellular membrane in different groups. Values are means±SE; n = 5 batches of cells. * P<0.05 vs. Ctrl.

**Figure 3:**
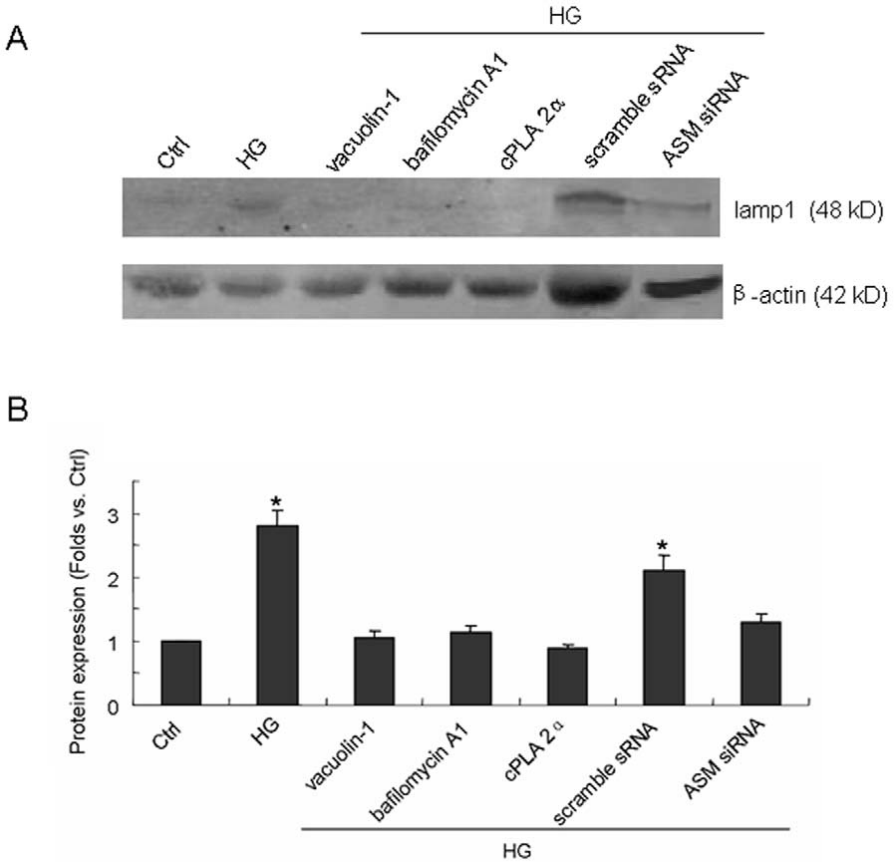
Effect of lysosomal inhibitors or ASM gene silencing on HG-induced increase of lamp1 protein quantity on cellular membrane. The proteins on cellular membrane were first isolated and then the lamp1 expression was detected by Western blot. A: representative band of lamp1 expression in cell incubated by normal glucose (Ctrl) or high glucose for 12 h (HG) with or without the pretreatment by vacuolin-1(10 µmol/l), bafilomycin A1 (100 nmol/l), cPLA 2α (20 µmol/l) for 1 h, or after scramble sRNA or ASM siRNA transfection. B: summarized data of lamp 1 protein quantity normalized by β-actin in different groups. Values are means±SE; n = 4 batches of cells. * P<0.05 vs. Ctrl.

Vacuolin-1 is the inhibitor of calcium dependent lysosomal moving and fusion, bafilomycin A1 would prevent the activity of vacuolar-type H^+^-ATPase of lysosome while cPLA 2α is a blocker of cytosolic phospholipase A2 which could keep lysosomal calcium from releasing into cytoplasm. In the present work, each of inhibitors was applied 1 h before and during 12 h incubation with normal or high glucose. As shown in the confocal image and summarized data of Figure 2, HG induced significant higher levels of lamp1 fluorescence on cellular membrane (4.55-fold, P<0.05), but in the vacuolin-1, bafilomycin A1 or cPLA 2α pretreated cell, HG did not change the lamp1 fluorescence as compared to that in Ctrl cell (0.88, 1.12 and 1.15-fold respectively) indicating HG-induced lysosome-membrane fusion could be prevented notedly by lysosomal inhibition. The figure 2 also showed that in scramble small RNA (sRNA) but not ASM siRNA transfected cell, HG could increase lamp1 fluorescence on cellular membrane remarkably (3.11 vs. 1.21-fold as compared to the control level, P<0.05) which means ASM siRNA transfection could block HG-induced lysosome-membrane fusion substantially. To confirm the outcome in confocal examination, we isolated the proteins on cellular membrane and then detected the lamp1expression by Western blot. As shown in Figure 3A, a single band at 48 kD was detected which was in accord with the expected size of unglycosylated lamp1. The Figure 3B showed that HG incubation increased the quantity of lamp1 protein on cellular membrane with 2.83-fold as compared to that in Ctrl cell (P<0.05), and the change could be blocked by the pretreatment of vacuolin-1, bafilomycin A1 or cPLA 2α which is analogy with the findings in confocal examination. And also as shown in confocal examination, in the scramble sRNA but not ASM siRNA transfected cell, HG increased the quantity of lamp1 protein on cellular membrane significantly (2.14 vs. 1.30-fold as compared to the control level, P<0.05).

### HG increased the activity of β-hexosaminidase and cathepsin C in cultural medium which was prevented by lysosomal inhibitors or ASM siRNA transfection

**Figure 4:**
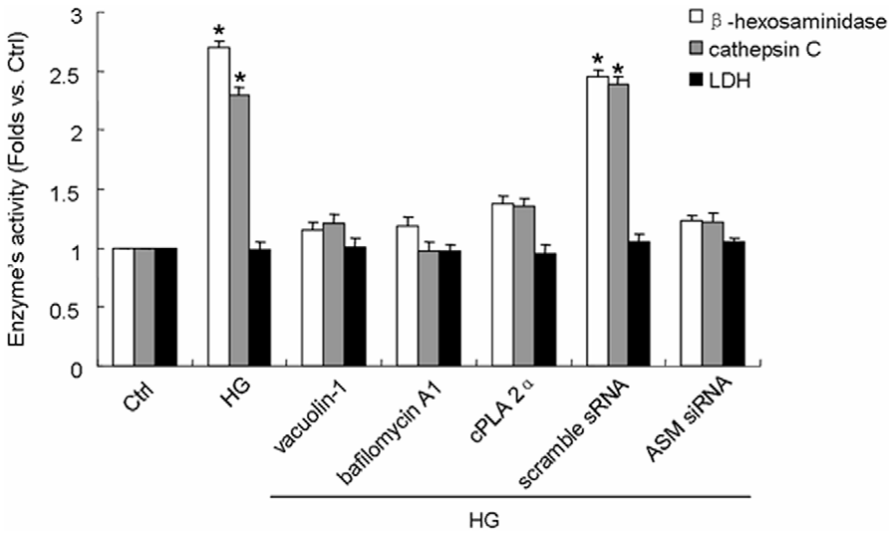
Effect of HG incubation with or without lysosomal inhibition or ASM gene silencing on the activity of β-hexosaminidase,cathepsin C or lactate dehydrogenase (LDH) in cultural medium. After incubation by normal glucose (Ctrl) or high glucose for 12 h (HG), the cultural medium was collected and the cells were lysed. The activity of β-hexosaminidase, cathepsin C and lactate dehydrogenase (LDH) were measured and the proportion of them in medium was calculated. Values are means±SE; n = 5 batches of cells. * P<0.05 vs. Ctrl.

To clarify HG-induced lysosome-membrane fusion further, we examined the activity of β-hexosaminidase, cathepsin C or lactic dehydrogenase (LDH) in cultural medium. β-hexosaminidase and cathepsin C are the symbolized enzymes of lysosome which could be transported into medium after lysosomal fusing to cellular membrane while LDH is located in cytoplasm and the increase of its concentration in medium indicates ruptured or dead cell. The ratio of enzyme activity detected in medium to the total enzyme activity detected in medium and cell lysate was calculated and compared among groups. Figure 4 showed that the activity of β-hexosaminidase and cathepsin C was increased by 170.3% and 131.5% respectively in HG incubated cell as compared to that in Ctrl cell. With pretreatment of vacuolin-1, bafilomycin A1 or cPLA 2α, HG incubation did not increase the activity of β-hexosaminidase or cathepsin C which means the transportation of lysosomal symbolized enzymes to cultural medium has been blocked by lysosmal inhibition. In the scramble sRNA but not ASM siRNA transfected cell, HG enhanced the activity of β-hexosaminidase and cathepsin C in cultural medium by 145.5% and 138.3% respectively indicating that ASM siRNA transfection could block HG-induced lysosome-membrane fusion substantially. On the contrary, the activity of LDH in all groups including HG, HG with pretreatment of vacuolin-1, bafilomycin A1 or cPLA 2α and HG with scramble sRNA or ASM siRNA transfection, did not show any significant difference as compared to that in Ctrl cell which means the cell was not ruptured or dead after 12 h incubation with HG.

### HG induced the production of ceramide in the cell which could be prevented by lysosomal inhibitors or ASM siRNA transfection

**Figure 5:**
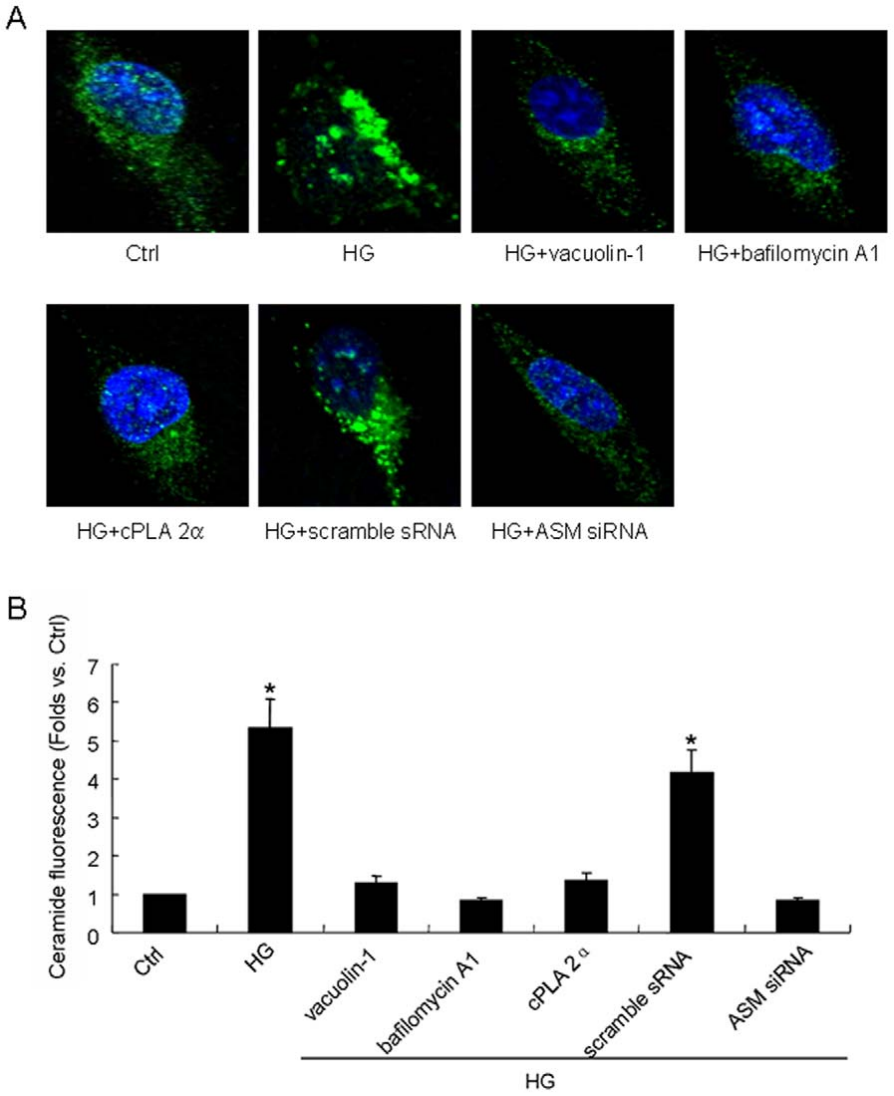
Effect of HG on ceramide fluorescence and the performance after lysosomal inhibition or ASM gene silencing. A: representative images of ceramide fluorescence in the cells which were cultured in normal glucose (5.6 mmol/l, Ctrl) or high glucose (22 mmol/l, HG) medium for 12 h with or without the pretreatment by vacuolin-1(10 µmol/l), bafilomycin A1 (100 nmol/l) or cPLA 2α (20 µmol/l) for 1 h or by the transfection of scramble small RNA (sRNA) or ASM small interfering RNA (siRNA). B: summarized results of ceramide fluorescent abundance in different groups. Values are means±SE; n = 5 batches of cells. * P<0.05 vs. Ctrl.

To examine the effect of HG alone or combined with lysosomal inhibition on ceramide generation in HUVEC, we incubated the cell with FITC labeled antibody for ceramide and detected the fluorescent signal by confocal microscope. As shown in the images (A) and summarized data (B) of Figure 5, in the Ctrl cell, ceramide presented as an uniform distribution, and not only could HG induce significant higher of ceramide fluorescence as compared to Ctrl (5.35-fold, P<0.05), but also it led to its clustering or aggregation around the cell. In the vacuolin-1, bafilomycin A1 or cPLA 2α pretreated cell, neither the fluorescent intensity nor the shape of ceramide could be changed by HG incubation indicating HG-induced ceramide production and aggregation were prevented significantly by lysosomal inhibition. The figure 5 also showed that in scramble sRNA but not ASM siRNA transfected cell, HG raised the ceramide fluorescence and induce ceramide clustering remarkably (4.15 vs. 0.82-fold as compared to the control level, P<0.05) which means ASM siRNA transfection could block HG-induced ceramide production substantially.

### HG promoted LRs clustering which were obstructed after inhibition of lysosome-membrane fusion

**Figure 6:**
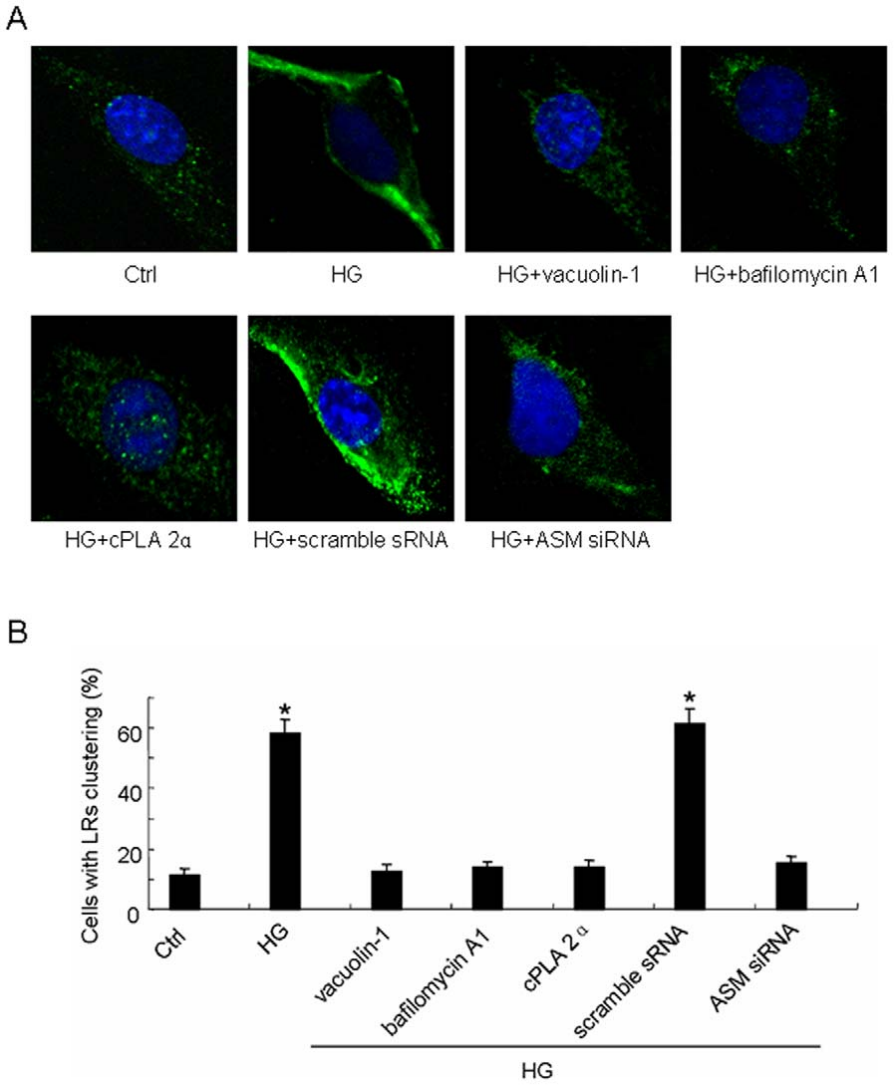
The effect of HG incubation on lipid rafts (LRs) clustering and the manifestations after inhibition of lysosome-membrane fusion. A: Representative images of LRs clustering in cell incubated by normal glucose (Ctrl) or high glucose for 12 h (HG) with or without the pretreatment by vacuolin-1(10 µmol/l), bafilomycin A1 (100 nmol/l), cPLA 2α (20 µmol/l) for 1 h, or after scramble sRNA or ASM siRNA transfection. B: summarized results on percentage of cells with LRs clusters in different groups. Values are means±SE; n = 5 batches of cells. * P<0.05 vs. Ctrl.

To detect the shape of LRs, the cells were labeled by Alexa Fluo 488 conjugated cholera toxin B (Al488-CTXB) and detected by confocal microscope. As shown in Figure 6A, under resting conditions (Ctrl), LRs were distributed over the cellular membrane by weak, diffused green fluorescence and HG incubation led to the formation of green Al488-CTXB labeled fluorescent patches in the cellular membrane which is the typical fluorescent microscopic images for LRs clustering and could also be found in HG incubated scramble sRNA transfected cell. Figure 6B summarized the effects of different treatments on such LRs clustering by counting positive cells with LRs clusters or patches. In Ctrl cell, only 11.5% of cells displayed positive LRs clustering, whereas 58.2% of cells displayed such LRs clustering after incubation with HG. HG with pretreatment of vacuolin-1, bafilomycin A1 or cPLA 2α did not change the percentage of positive cell significantly as compared to that in Ctrl cell which means the HG-induced LRs clustering was blocked after inhibition of lysosome-membrane fusion. Also, in the scramble sRNA transfected cell, HG increased percentage of cell with LRs clusters which was not present in ASM siRNA transfected cell indicating the interference of ASM expression could prevent HG-induced LRs clustering remarkably.

### HG facilitated the colocalization of gp91*^phox^* to LRs clusters which was blocked by lysosome-membrane fusion inhibition

**Figure 7:**
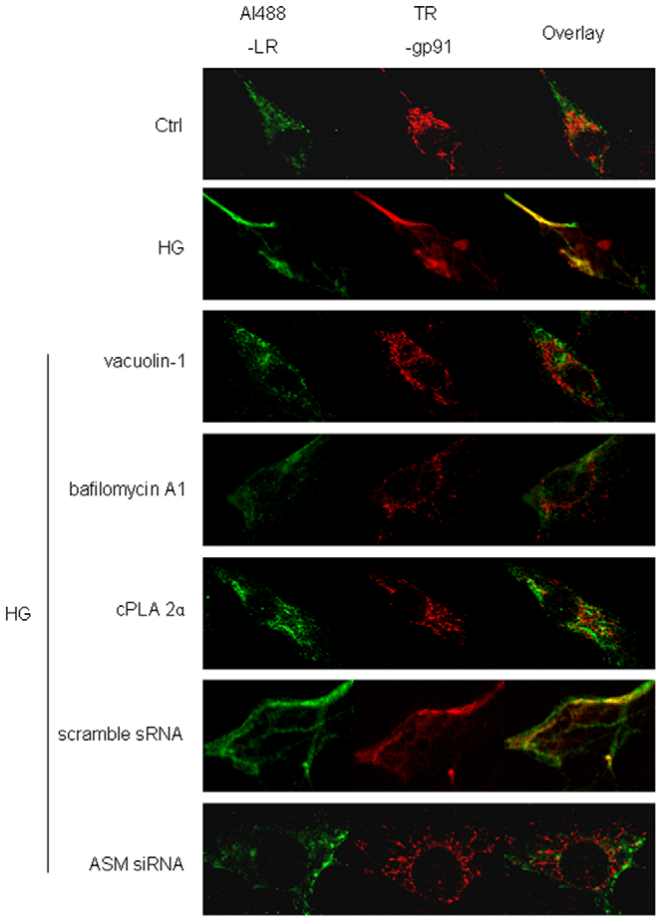
Effects of HG incubation on the colocalization of gp91*^phox^* to LRs and the representations after inhibition of lysosome-membrane fusion. Representative confocal images showed Alexa Fluo 488 conjugated cholera toxin B labeled LRs clusters (Al488-LR) and Texas red labeled gp91*^phox^* (TR-gp91) in cell incubated by normal glucose (Ctrl) or high glucose for 12 h (HG) with or without the pretreatment by vacuolin-1(10 µmol/l for 1 h), bafilomycin A1 (100 nmol/l for 1 h), cPLA 2α (20 µmol/l for 1 h), scramble sRNA transfection or ASM siRNA transfection. The overlay images exhibited yellow spots or patches (right), which represented colocalization of gp91*^phox^* with LRs component, ganglioside G_M_1.2. The figure showed representative images from experiment of 5 batches of HUVEC.

**Figure 8:**
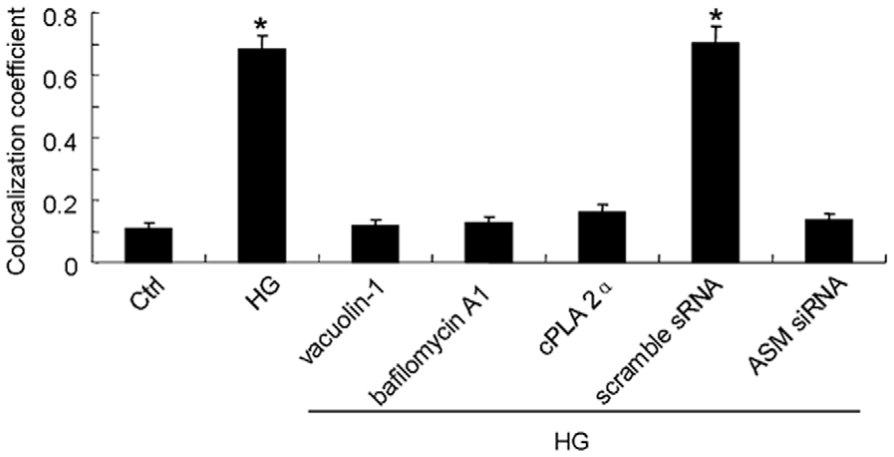
Summarized data on the colocalization of LRs with gp91*^phox^* in different groups. HG incubation increased the proportion of LRs colocalized by gp91*^phox^* significantly in normal or scramble sRNA transfected cell. But in vacuolin-1, bafilomycin A1 or cPLA 2α pretreated and ASM siRNA transfected cell, HG incubation did not change the level of colocalization between LRs and gp91*^phox^* as compared to that in Ctrl cell. Values are means±SE; n = 5 batches of cells. * P<0.05 vs. Ctrl.

It was reported that NOX localization in LRs clusters is an important feature of LRs-redox signaling platforms. As shown in the confocal images in Figure 7, fluorescent patches were identified by Al488-CTXB (green) and anti-gp91*^phox^* antibody, plus a Texas red secondary antibody (red). Yellow patches or dots in the overlaid image showed colocalization of gp91*^phox^* in LR clusters. We demonstrated that Ctrl cell displayed only some diffuse staining of both fluorescents and just tiny amount of colocalization of them whereas HG stimulated the translocation of gp91*^phox^* to LRs clusters substantially. HG with predisposal of vacuolin-1, bafilomycin A1 or cPLA 2α did not change the percentage of gp91*^phox^* colocalized LRs as compared to that in Ctrl cell which means HG-induced colocalization of gp91*^phox^* to LRs has been restored by inhibition of lysosome-membrane fusion. Also, scamble sRNA but not ASM siRNA transfected cell could be stimulated by HG to show translocation of gp91*^phox^* to LRs clusters. The summarized data in Figure 8 showed that the colocalization of LRs with gp91*^phox^* after HG incubation was significantly higher than that in Ctrl cell (0.68±0.05 vs. 0.11±0.02, P<0.05). HG with pretreatment of vacuolin-1, bafilomycin A1, cPLA 2α did not lead to higher percentage of gp91*^phox^* colocalization with LRs as compared to control level. In scramble sRNA but not ASM siRNA transfected cell, HG incubation promoted proportion of LRs colocalized by gp91*^phox^* significantly.

### HG increased superoxide (O_2_^.^^-^) production and nitric oxide (NO) quenching which were prevented by lysosome-membrane fusion inhibition or LRs disruption

**Figure 9:**
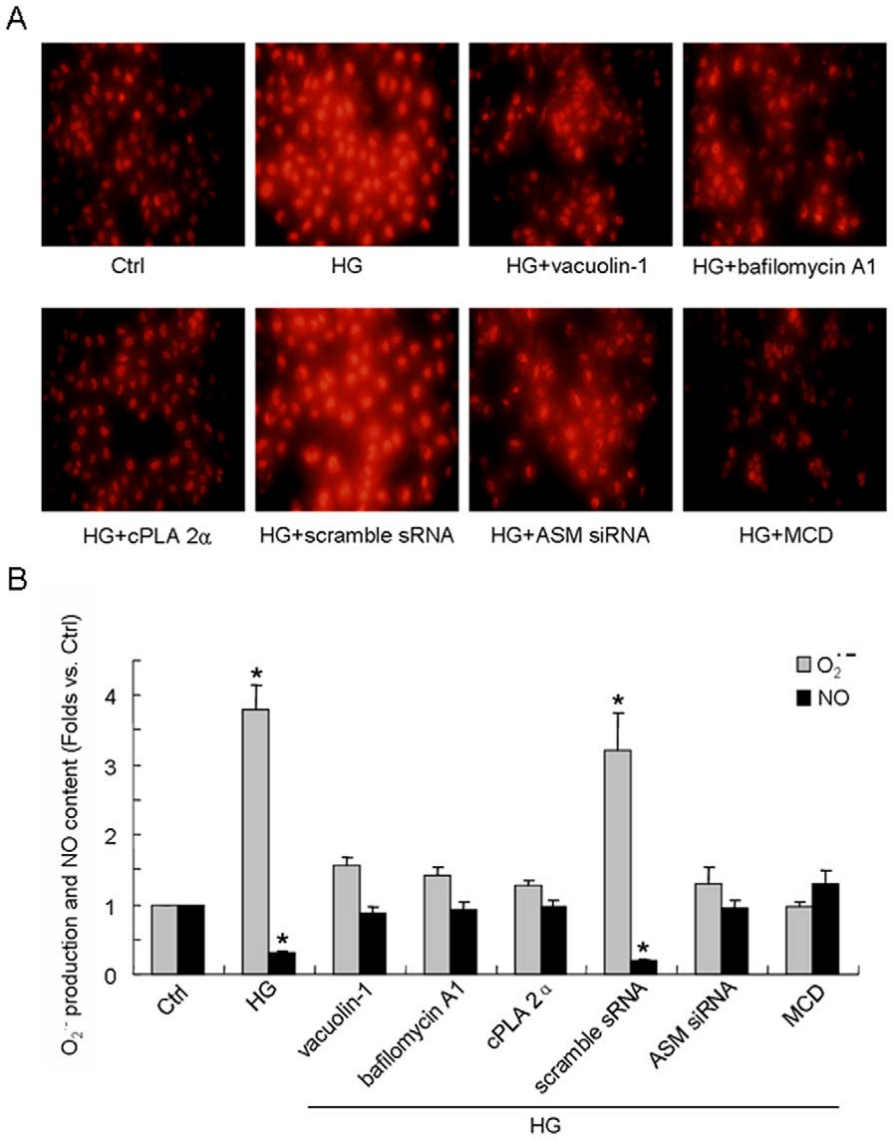
Effects of lysosome-membrane fusion inhibition or LRs disruption on HG-induced oxidative stress formation and nitric oxide (NO) quenching. The superoxide (O_2_^.^^-^) formation was detected with a DHE probe and the NO content was measured by nitrate reductase method. A: Representative staining of superoxide (O_2_^.^^-^) production in cell incubated in normal glucose (Ctrl) or high glucose for 12 h (HG) with or without the pretreatment by vacuolin-1(10 µmol/l for 1 h), bafilomycin A1 (100 nmol/l for 1 h), cPLA 2α (20 µmol/l for 1 h), scramble sRNA transfection, ASM siRNA transfection or methylcyclodextrin (MCD, 10 mmol/l for 1 h). B: summarized results on O_2_^.^^-^ production and NO content in different groups. Values are means±SE; n = 5 batches of cells. * P<0.05 vs. Ctrl.

To evaluate the effect of HG incubation on oxidative stress and NO content of cell, a dihydroethidium (DHE) fluorescence probe was used and a nitrate reductase method was applied respectively. In DHE-loaded cell, HG incubation enhanced the O_2_^.^^-^ generation by 3.57-fold as compared to that in Ctrl cell (P<0.05) which was blocked by pretreatment of vacuolin-1, bafilomycin A1 or cPLA 2α (Figure 9A and Figure 9B). Scramble sRNA but not ASM siRNA transfected cell could also be stimulated to produce O_2_^.^^-^ remarkably by HG incubation (3.18-fold, P<0.05). As we have known, O_2_^.^^-^ reacts rapidly with NO producing peroxynitrite and depleting NO bioactivity which plays a potentially important role in the pathogenesis of endothelial dysfunction. So we also investigated whether HG incubation have a direct effect on NO content of the cell. As shown in Figure 9B, corresponding to O_2_^.^^-^ production, HG led to the quenching of NO showing as 0.27-fold of decrease of its content (P<0.05). Besides, pretreatment of vacuolin-1, bafilomycin A1 or cPLA 2α and ASM siRNA transfection, but not scramble sRNA transfection could restore the HG-induced NO breakdown. Furthermore, methylcyclodextrin (MCD), a drug that depletes cholesterol from the membrane and leads to LRs disruption, was applied and as shown in Figure 9, it could also reverse the HG-induced O_2_^.^^-^ production and NO quenching to the level statically non-significant than that in Ctrl cell.

### Lysosome fusion inhibition or LRs disruption reversed HG-induced impairment of endothelium-dependent NO-mediated vasodilation

**Figure 10:**
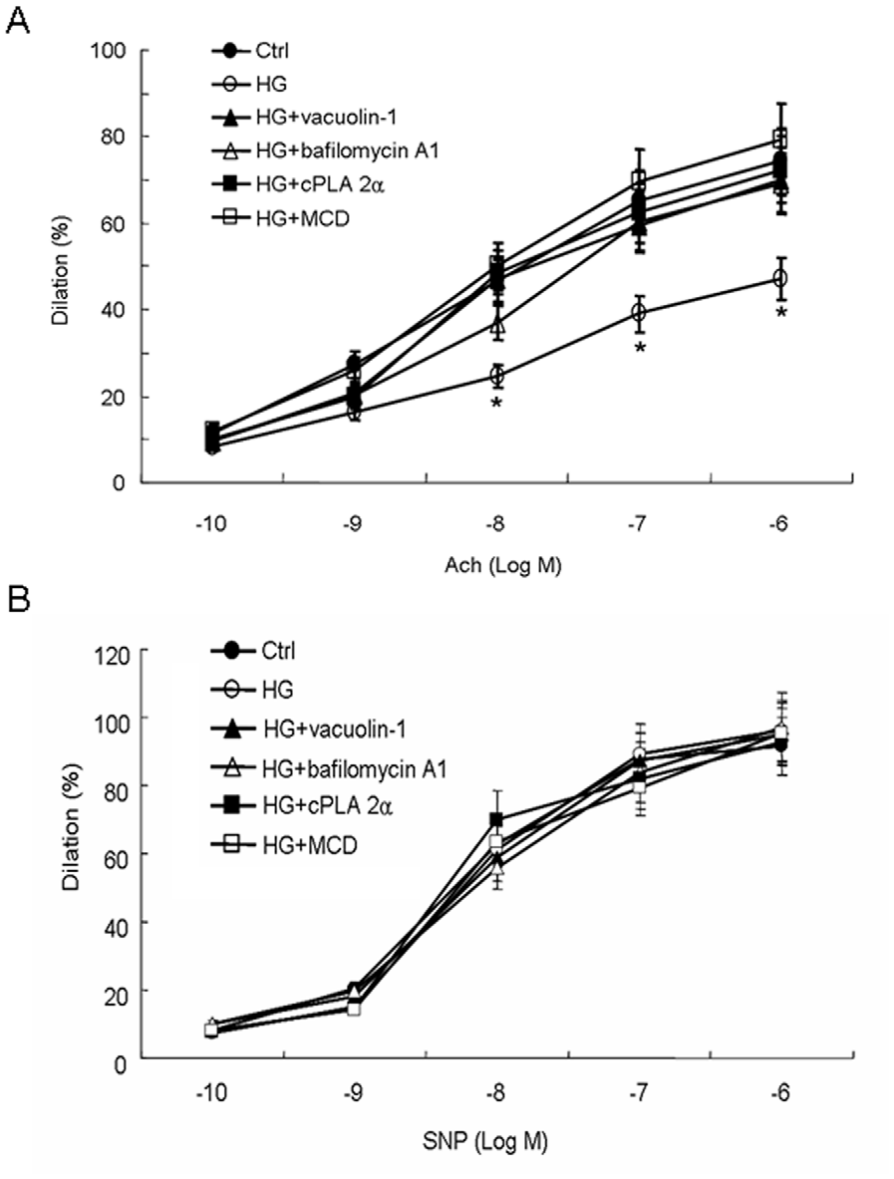
Effect of normal (5.6 mmol/l, Ctrl) or high glucose (22 mmol/l, HG) incubation with or without pretreatment by lysosomal inhibitors or MCD (10 mmol/l for 1 h) on acetylcholine (Ach)-induced NO-mediated endothelium-dependent vasodilation (A) or sodium nitroprusside (SNP)-induced endothelium-independent vasodilation (B) in isolated rat abdominal aortas. Ach-induced NO-mediated endothelium-dependent vasodilation was impaired significantly by HG incubation (open circles) at Ach concentration of 10^−8^ mol/l, 10^−7^ mol/l and 10^−6^ mol/l as compared with Ctrl (black circles), and the effect was restored by pretreatment of vacuolin-1 (black triangles), bafilomycin A1 (open triangles), cPLA 2α (black squares) or MCD (open squares). SNP-induced NO-mediated endothelium-independent vasorelaxation did not differ among groups including Ctrl, HG, and HG with pretreatments mentioned above. Values are means±SE; n = 5 times of assay. * P<0.05 vs. Ctrl at the same concentration of Ach.

To investigate the effects of high glucose on vascular reactivity, isolated rat abdominal aortas were cultured in medium of normal (5.6 mmol/l, Ctrl) or high glucose (22 mmol/l, HG) for 12 h with or without 1h pretreatment by vacuolin-1, bafilomycin A1 or cPLA 2α or MCD and then were mounted in a tissue-organ bath system. Endothelium-dependent NO-mediated vasodilation was tested in these arteries during precontraction with high potassium (65 mmol/l) in the presence of 10 µmol/l indometacin. As shown in Figure 10A, HG incubation reduced acetylcholine (Ach)-induced vasodilation significantly by 19.4%, 31.7%, 36.5% at Ach concentration of 10^−8^ mol/l, 10^−7^ mol/l and 10^−6^ mol/l respectively, and that were reversed to the level non-significant with Ctrl by predisposal of vacuolin-1, bafilomycin A1, cPLA 2α or MCD. The effect of HG was not due to changes in osmotic values for the osmotic control mannitol incubated aortas displayed normal relaxing responses to Ach (data not shown). The effect of HG, lysosome-membrane fusion inhibitors or MCD was strictly endothelium-dependent, because no alterations in endothelium-independent relaxation were provoked with the NO-donor SNP (Figure 10B).

## Discussion

In this study HG incubation has been shown to induce lysosomal fusion to cellular membrane in HUVEC which promoted ceramide generation, LRs clustering and translocation of gp91*^phox^* to LRs clusters, then facilitating the O_2_^.^^-^ production and NO quenching or depletion. Functionally, the endothelium-dependent NO-mediated vasodilation of aorta was blunted by HG incubation. Furthermore, we found that HG-induced ceramide generation, LRs clustering, O_2_^.^^-^ production and endothelial dysfunction were prevented after lysosome-membrane fusion was inhibited or LRs was disrupted both before and during HG stimulation. So the work indicates that the lysosomal fusion and the following LRs clustering play a crucial role in the endothelial impairment induced by HG incubation.

Traditionally, lysosomes are membrane-bound organelles that contain acid hydrolases and degrade macromolecular coming from the secretory, endocytic, autophagic and phagocytic membrane-trafficking pathways. However, the function of lysosomes has been found much more than degradation of protein, lipids, nucleic acid or carbohydrate [8], [9]. In secretory cell, specialized lysosomal compartments that store newly synthesized secretory proteins are referred to as secretory lysosomes or lysosome-related organelles. The melanosomes, class II major histocompatibility complex compartments, basophil granules, neutrophil azurophil granules, platelet-dense granules, mast-cell secretory granules, eosinophil-specific granules, cytotoxic T lymphocyte lytic granules and ATP or glucagons containing granules have been classified to it [8]–[12]. Morphological and biochemical studies reveal that these ‘secretory lysosomes’ combined many of the characteristics of conventional lysosomes and secretory granules into a single structure [13]. While in non-secrectory cell, lysosomes can also undergo fusion with plasma membrane functioning for repairing plasma membrane damage [14]–[18] and disposing of surplus membrane accumulated by lysosomes [13]. Lysosome-membrane fusion has also been found after exposure to stress signals such as irradiation, heat shock, UV light exposure, or bacterial infection [25]. In addition, lysosomal recruitment to the plasma membrane seems to play a role in infection by some intracellular parasites. For example, *T. cruzi* invasion may take advantage of lysosomal fusion with the plasma membrane as a mode of entry to the cell [8], [9], [13], [19].

Recently, lysosome-membrane fusion has been detected in bovine coronary arterial endothelial cells and glomerular endothelial cells especially when the cells encountered with apoptotic stimuluses such as FasL, tumor necrosis factor (TNF)-α, endostatin or homocysteine which has been shown to participate in the ROS production and elicit the endothelial dysfunction [7], [20], [21], [22]. But whether the lysosome-membrane fusion could also happen in other types of endothelium such as HUVEC and be caused by hyperglycaemia or diabetes are, as yet, largely unclear, that we investigated from multiple aspects in the present work. We first detected the expression of lamp1, EEA-1 and Rab-7, which are the marker of lysosome, early endosome and late endosome respectively, on membrane of HUVEC after HG incubation for different length of time. In the cell incubated by HG for 6 h, the marker of lysosome but not endosomes began to appear significantly in cellular membrane which reached to the maximum after 12 h stimulation. The reason we detected endosomes in the work is to clarify the “extra” membrane on cellular membrane is from lysosomal moving outside and fusion, but not from endosomal recycling. Apart from morphological examination, we also measured the quantity of lamp1 protein in cellular membrane extraction and the activity of lysosomal symbolized enzymes in cultural medium. Both of assays have shown very consistent result which supported our hypothesis further. It also proved that incubation in 22 mmol/l of glucose for 24 h could not lead to cell death or rupture. To our knowledge, it is the first work indicating HG incubation could induce lysosomal fusion to cellular membrane in endothelium. According to the previous studies, the increased [Ca^2+^]_i_ we found in the present work (Figure 1) which has also been reported by others [23] may act as the impelling force for the lysosomal moving and fusion observed [8]–[11], [14]–[19]. Another possible cause for HG-induced lysosome-membrane fusion may be the reported needle-like injury of cell membrane in hyperglycaemia [24] for the fused lysosomal membrane could be used as patches for the wound repair.

Three inhibitors were used in the present work to block lysosome-membrane fusion in different ways. Among them vacuolin-1 can induce rapid formation of large, swollen structures derived from lysosomes by homotypic fusion which is usually applied as a blocker for both ionophore- and wound-induced, calcium-dependent exocytosis of lysosomes [26]. Bafilomycin A1 is a specific inhibitor of the vacuolar type H^+^-ATPase (V-ATPase) in cells which could prevent the lysosomal acidification and reduce the calcium content in lysosome [27]. While cPLA 2α is the cytosolic phospholipase A_2_ inhibitor which has been found decrease the lysosomal calcium release and hinder its moving remarkably [28]. We also carried out siRNA transfection to downregulate the expression of ASM, a lysosomal glycoprotein catalyzing the degradation of membrane-bound sphingomyelin into phosphocholine and ceramide [20], [29], [30]. By generation of ceramide, the ASM could enhance the fusogenicity of cellular membranes at the intra- or intercellular level and induce the membrane proximal lysosome fusion into the cell membrane in endothelial cells [7], [31]. Although ASM seems not to be critical for the generation of the fusion pore per se, it contributes to the widening of the initial fusion, as would be required for the merging of lysosomes with a phagosome in macrophages. Under this condition, ceramide is confined to the membranes of phagosomes and lysosomes or the extracellular leaflet of the plasma membrane. It has also been suggested that ASM regulates vesicular fusion processes by modifying the steric conformation of cellular membranes [7], [31]. In the present work, as expected, both the lysosomal functional inhibitors and ASM siRNA transfection have been shown to be able to prevent HG-induced lysosome-membrane fusion significantly.

Lipid rafts (LRs) that consist of dynamic assemblies of cholesterol, lipids with saturated acyl chains, such as sphingolipids and glycosphingolipids in the exoplasmic leaflet of the membrane bilayer, are now emerging as an important cellular signaling mechanism in the regulation of a variety of cellular functions [32], [33], [34]. It has been reported that LRs normally exist in dispersed state and would be clustered under apoptotic stimulus and form relatively large macrodomains. The LRs clustering may serve to recruit or aggregate various receptors, such as tumor necrosis factor (TNF)-α receptors, insulin receptors, or Fas, and also aggregate various signaling molecules, such as trimeric G proteins, sphingomyelin, tyrosine kinases, and phosphatases, resulting in activation of different signaling pathways [5], [6], [35], [36]. As far as the mechanisms for the dispersed LRs to be clustered, lysosomal moving and fusion to cell membrane has been reported to be crucial. The cause lies in the fact that it would transport ASM, which is mainly located in lysosomes, onto the extracellular leaflet of the cell membrane in contact with its substrate sphingomylin, one of the most abundant lipids in the outer layer of the plasma membrane [7], [29], [30]. And then the ASM-mediated hydrolytic removal of the phosphorylcholine head group of sphingomyelin take place which generates extracellularly oriented ceramide on the plasma membrane [37], [38]. In light of its biochemical and biophysical properties, ceramide molecules tend to associate with each other and form ceramide-enriched microdomains that spontaneously fuse to large ceramide enriched membrane platforms [5], [6], [22], [35], [37], [38] and provide a driving force for the LRs clustering. In our study, HG incubation induced the lysosome-membrane fusion promoting not only the ceramide production but also the aggregation of it presented as relative large patches around cell (Figure 5) which, according to the paper review above, may lead to the LRs clustering as shown in Figure 6 and 7.

NADPH oxidase is an important redox-signaling enzyme in endothelial cells and its activation importantly relies on the assembling and aggregation of several subunits, including its membrane-associated subunits gp91*^phox^* or its isoforms and p22*^phox^*, as well as cytosolic subunits p47*^phox^*, p40*^phox^*, p67*^phox^*, and Rac which could be mediated by LRs clustering and ceramide-enriched membrane platforms formation [4], [5], [21], [39], [40]. Hyperglycaemia incubation is also a potent stimulus for NADPH oxidase activation and O_2_^.^^-^ production, our results showed that it could elicit the lysosomal fusion, LRs clustering, LRs-NOX platform formation, O_2_^.^^-^ production and finally result in endothelial dysfunction. Together with the report that the injurious effect of visfatin, an adipokine involved in the development of various obesity-associated pathologies, is associated with the formation of LRs-redox signaling platforms and local oxidative stress production via LRs clustering [41], we may be able to regard the process as a general pathway for the ROS production and endothelial damage under different pathological situations.

What are the clinical implications of these observations? Because high plasma level of ASM has been demonstrated in many pathological situations including chronic heart failure, atherosclerosis, hypertension, senescence and alcohol-dependence, which may be induced by lysosomal exocytosis in endothelial cells where ASM is primarily located [42], [43], [44]. A more recent study shows that dynamic modification of sphingomyelin in lipid microdomains controls development of obesity, fatty liver, and type 2 diabetes [45] which is also related to the ASM transportation by lysosomal exocytosis and fusion to cellular membrane. So we propose here that lysosome-membrane fusion plays a pivotal role in the impairment of endothelium-dependent vasodilation and development of vascular complications. Furthermore, prevention of lysosomal exocytosis or fusion may become a new target to delay or prevent vascular injury in diabetes from happening, especially at the early stage.

In summary, we found that HG incubation promote lysosome-membrane fusion in HUVEC, which was triggered by increased intracellular calcium concentration and mediated by ASM activation. Through LRs clustering and LRs-NOX platforms formation, oxidative stress was produced and NO was quenched and finally, the endothelium-dependent NO-mediated vasorelaxation was impaired. These data provide a new mechanistic link between hyperglycaemia, lysosome-membrane fusion, LRs clustering and vascular complications.

## Materials and Methods

### Ethics statement

All animal studies were carried out in accordance with the Guide for the Care and Use of Laboratory Animals of the National Institutes of Health. All experiments involving rats were reviewed and approved by the Ethics Committee for animal care and use of Fourth Military Medical University, P.R. China (Permit Number: 10010).

### Endothelial cell culture and treatments

The human umbilical vein endothelial cell lines (HUVEC) were purchased from ATCC (ATCC Number CRL-1730™) and cultured in DMEM (Invitrogen, Carlsbad, CA, USA) containing 10% fetal bovine serum (HyClone, Waltham, MA, USA), 100 units/ml penicillin, 100 mg/ml streptomycin, and 5 units/ml heparin [46] in the presence of normal glucose (Ctrl, 5.5 mM), high glucose (HG, 22 mmol/l), or 5.5 mM glucose plus 16.5 mM mannitol (mannitol) and maintained at 37°C in 5% CO_2_/95% air. As needed, one of the inhibitors of lysosomal movement including vacuolin-1 (10 µmol/l, Sigma, St. Louis, MO, USA), bafilomycin A1 (100 nmol/l, Sigma, St. Louis, MO, USA) or cPLA 2α (20 µmol/l, Sigma, St. Louis, MO, USA) was applied 1 h before and during glucose incubation. All studies were performed by using HUVEC prior to passage five.

### Measurement of intracellular free calcium concentration ([Ca^2+^]_i_)

The [Ca^2+^]_i_ of HUVEC was measured with the fluorescence Ca^2+^ indicator dye Fluo-3. To be loaded with Fluo-3, a glass coverslip with cultured cells was incubated with 5 µmol/l Fluo-3/acetoxymethyl ester (Fluo-3/AM, Molecular Probes, Carlsbad, CA, USA) for 30 min at 37°C and then rinsed with fresh DMEM. Consequently, the fluorescence was scanned under a laser-scanning confocal microscope (Olympus FV1000, Olympus Corporation, Tokyo, Japan) equipped with the FV10-ASW system. Fluo-3 in cells was excited at 488 nm, and emitted fluorescence was measured at >505 nm and fluorescence images were acquired. Further image manipulation or analysis was carried out using FV10-ASW system which could provide the fluorescent intensities of specific area. The average fluorescence of randomly selected 50 cells was calculated by two independent observers and the results were expressed as the fold changes of control.

### Indirect cellular surface immunofluorescent labeling

Following treatments, HUVEC on glass coverslips were washed several times with chilled PBS and incubated with monoclonal antibody against lamp1 (1∶200 in PBS/1% BSA, Sigma, St. Louis, MO, USA), EEA-1 (1∶100 in PBS/1% BSA, Sigma, St. Louis, MO, USA) or Rab-7 (1∶100 in PBS/1% BSA, Sigma, St. Louis, MO, USA) for 30 min on ice. After washing, the cells were incubated with Alexa 488-conjugated anti-mouse antibody (1∶1000, Molecular Probes, Carlsbad, CA, USA) and then were fixed with 4% formaldehyde for 30 min. The immunofluorescent labelings were visualized on an Olympus FV1000 microscope and images were acquired by using FV10-ASW system. The average fluorescence around cellular membrane in randomly selected 50 cells was calculated by two independent observers and the results were expressed as the fold changes of control.

### RNA interference

The ASM small interfering RNA (siRNA) is designed and verified by Santa Cruz Biotechnology (sc-41650, Santa Cruz, CA, USA). The scramble small RNA (sRNA) is also purchased from Santa Cruz Biotechnology (sc-36869, Santa Cruz, CA, USA) which has been confirmed as non-silencing double stranded RNA and was used in the present study to show the efficiency of transfection and act as an internal control. The transfection of ASM siRNA and scramble sRNA was performed using a QIAGEN TransMessenger transfection kit (QIAGEN, Valencia, CA, USA) according to the instruction manual.

### Enzyme's activity assays

HUVEC were grown in six-well plates (Corning Costar Corporation, Cambridge, MA, USA). Following treatment, medium was first collected, then detached cells and cellular debris were spun down in a microfuge at 1000 *g* for 5 min. After lysis of the cell in PBS containing 0.5% (v/v) Triton X-100 and centrifugation at 13,000 *g* for 5 min, the supernatant was got for the following test. The activity of cathepsin C and β-hexosaminidase in medium and cell lysates were assayed by commercial enzyme-linked immunosorbent assay (ELISA) kits (R&D Systems, Minneapolis, MN, USA) according to the instruction manual. The optical density was recorded using a micro-plate reader (µQuant; Bio-Tek Instruments, VT, USA) at 450 nm. The activity of lactic dehydrogenase (LDH) in medium and lysates was assayed with a commercial kit using acetone acid dinitro phenylhydrazone as a color indicator (Jiancheng Bioengineering Institute, Nanjing, China). Measurements were corrected for dilutions and results are expressed as the ratio of enzyme activity detected in medium to the total enzyme activity detected in medium and cell lysates. Means of triplicate results were compared among groups and the data was shown as the fold changes of control.

### Cell surface protein isolation and Western blot

The protein on cellular membrane was isolated by using a cell surface protein isolation kit (Pierce Biotechnology, Rockford, IL, USA). The cells were first labeled by biotinylation and then lysated through sonicating on ice. After combination on the column filled with the NeutrAvidin agarose slurry, the protein was eluted and collected. Then 50 µg of protein was separated by using 10% SDS-PAGE. After transferring the protein from gel to PVDF membrane, the target protein was detected with monoclonal antibody against lamp1 (1∶1000, Sigma, St. Louis, MO, USA) followed by HRP-conjugated anti-mouse antibody (Pierce Biotechnology, Rockford, IL, USA). The fluorescent band was detected by a Gel Image Analyzing System (Tanon-4200, Tanon Science & Technology, Shanghai, China).

### Confocal microscopic analysis of ceramide production, LRs clustering and colocalization of gp91*^phox^* to LRs clusters in HUVECs

The HUVECs grown on glass coverslips were fixed with 4% paraformaldehyde in PBS for 10 min. For just detect the protein expression on cellular membrane, no permeabilization was done in the study. For the examination of ceramide production, the cells were first incubated with mouse anti-ceramide monoclonal antibody (1∶100, Sigma, St. Louis, MO, USA) followed by FITC-conjugated anti-mouse secondary antibody (1∶200, Abcam, Cambridge, MA, USA). lipid rafts (LRs) were detected with Alexa Fluo 488 conjugated cholera toxin B (Al488-CTXB, 2 µg/ml, 2h, Molecular Probes, Carlsbad, CA, USA) under a conventional Zeiss fluorescence microscope or a Olympus FV1000 confocal microscope. The patch formation of Al488-CTXB labeled gangliosides complex represented the clusters of LRs. In each experiment, the presence or absence of clustering in samples of 200 cells was scored by two independent observers. The results were given as the percentage of cells showing clusters after the indicated treatment. For dual-staining detection of the colocalization of LRs with gp91*^phox^*, the cells were first incubated with Al488-CTXB, and then with mouse anti-gp91*^phox^* monoclonal antibody (1∶75, Abcam, Cambridge, MA, USA), which was followed by Texas red-labeled anti-mouse secondary antibody (1∶200, Abcam, Cambridge, MA, USA). Then the colocalizations were visualized with confocal microscopy, the images were acquired by using FV10-ASW system and then analyzed by using the Image Pro Plus 6.0 software (Media Cybernetics, Bethesda, MD, USA). The colocalization coefficient was represented by Pearson's correlation coefficient.

### Superoxide (O_2_^.^^-^) production and Nitric oxide (NO) content measurement

The O_2_^.^^-^ content was measured with the dihydroethidium (DHE) fluorescence probe (Invitrogen, Carlsbad, CA, USA). After treatment, the cells grown on glass coverslips were loaded with 50 µmol/l DHE for 30 min, and then the image was acquired by using Olympus FV1000 confocal microscope and analyzed with FV10-ASW system. A commercial kit was used (Jiancheng Bioengineering Institute, Nanjing, China) to examine the NO content in HUVEC. The cells were first collected into lysis buffer (320 mmol/l sucrose, 1 mmol/l EDTA, 1 mmol/l DTT, 10 µg/ml leupeptin, 2 µg/ml aprotinin, pH 7.4) and then was sonicated and homogenized on ice. After centrifugation at 4°C, the supernatant was collected for NO content measurement by nitrate reductase method according to the instruction manual and for protein concentration assay by using a BCA protein assay kit (Pierce Biotechnology, Rockford, IL, USA). The NO content normalized by protein concentration was calculated. The content of both O_2_^.^^-^ and NO were shown as the fold changes of control.

### Isolated abdominal aorta tension recording

The abdominal aorta of Sprague Dawley rats were dissected and stored in cell culture medium. After incubation in medium of normal glucose (Ctrl, 5.5 mM) or high glucose (HG, 22 mmol/l) for 12 h with or without predisposal of vacuolin-1 (10 µmol/l, 1h, Sigma, St. Louis, MO, USA), bafilomycin A1 (100 nmol/l, 1h, Sigma, St. Louis, MO, USA), cPLA 2α (20 µmol/l, 1h, Sigma, St. Louis, MO, USA) or methylcyclodextrin (MCD, 10 mmol/l, 1 h, Sigma, St. Louis, MO, USA), the artery was mounted in a Radnoti High-Tech Tissue-Organ Bath System and the isometric wall tension was recorded by a PowerLab Data Acquisition Systems (AD Instruments, Bella Vista NSW, Australia). After 30 min of equilibration in physiological saline solution (pH 7.4) containing (in mmol/L): 119 NaCl, 4.7 KCl, 1.6 CaCl_2_, 1.17 MgSO_4_, 1.18 NaH_2_PO_4_, 2.24 NaHCO_3_, 0.026 EDTA, and 5.5 glucose, at 37°C, bubbled with a gas mixture of 95% O_2_ and 5% CO_2_, basic tension was set. Then the arteries were precontracted with 65 mmol/l K^+^ (which blocks endothelium derived hyperpolarizing factors), following preincubation with the prostaglandin blocker indometacin (10^−5^ mol/l, Sigma, St. Louis, MO, USA). Once steady-state contraction was obtained, cumulative dose-response curves to the endothelium-dependent vasodilator acetylcholine (Ach, 10^−10^ to 10^−6^ mol/l, Sigma, St. Louis, MO, USA) or endothelium-independent vasodilator sodium nitroprusside (SNP, 10^−10^ to 10^−6^ mol/l, Sigma, St. Louis, MO, USA) were determined by measuring changes in wall tension.

### Statistical analysis

Data are presented as mean±SEM. Significant differences between and within multiple groups were examined by using ANOVA for repeated measures, followed by Duncan's multiple-range test. A value of *p*<0.05 was considered statistically significant.
